# In Situ Growth of Au NPs on Nitrogen-Doped Graphene Quantum Dots Decorated Graphene Composites for the Construction of an Electrochemical Immunosensor and Its Application in CEA Detection

**DOI:** 10.3390/molecules30061347

**Published:** 2025-03-17

**Authors:** Zhengzheng Yan, Lujie Wang, Fei Yan

**Affiliations:** 1 Shanxi Bethune Hospital, Shanxi Academy of Medical Sciences, Tongji Shanxi Hospital, Third Hospital of Shanxi Medical University, Taiyuan 030032, China; yanzhengzheng@sxmu.edu.cn; 2 Key Laboratory of Surface & Interface Science of Polymer Materials of Zhejiang Province, Department of Chemistry, School of Chemistry and Chemical Engineering, Zhejiang Sci-Tech University, Hangzhou 310018, China; 202220103145@mails.zstu.edu.cn

**Keywords:** Au nanoparticles, nitrogen-doped graphene quantum dots, reduced graphene oxide, electrochemical immunosensor, carcinoembryonic antigen

## Abstract

Carcinoembryonic antigen (CEA) is an important tumor biomarker for the early clinical diagnosis of various cancers, and, therefore, the accurate and sensitive quantitative determination of CEA is of vital significance. In this study, we demonstrated the in situ growth of Au nanoparticles (AuNPs) on nitrogen-doped graphene quantum dots (N-GQDs) decorated reduced graphene oxide (rGO) nanocomposites by using simple drop-coating and electrochemical deposition methods. N-GQDs@rGO can be formed through the π–π stacking interaction and possesses a high specific surface area and many functional groups, providing lots of anchor sites (amino moieties in NGQDs) for the in situ electrochemical growth of AuNPs without the addition of reductants and protective agents. Such AuNPs/N-GQDs@rGO ternary nanocomposites combine the characteristics of three nanomaterials, showing a large surface area, excellent solubility, good conductivity, catalytic activity, a simple fabrication process, and notable stability, which are further used to construct a label-free electrochemical immunosensor for the determination of CEA. Under the optimized experimental conditions, the AuNPs/N-GQDs@rGO-based electrochemical immunosensor achieves a broad linear response, ranging from 1 pg/mL to 0.5 μg/mL and a low detection limit of 0.13 pg/mL. Moreover, the AuNPs/N-GQDs@rGO-based electrochemical immunosensor shows exceptional selectivity, anti-interference, and anti-fouling capabilities for the direct analysis of CEA amounts in fetal bovine serum samples, showing vast potential in the clinical screening of cancer.

## 1. Introduction

Cancer is a widespread disease which poses significant treatment challenges, and is characterized by persistently high mortality rates [1]. Therefore, effective early diagnosis is crucial for improving survival rates and managing disease progression. Tumor marker levels in the serum of cancer patients are often significantly elevated, reflecting tumor status and providing essential information for diagnosis, classification, treatment selection, and prognosis [2,3]. Carcinoembryonic antigen (CEA) is a commonly used tumor marker, which has been extensively applied in the clinical diagnosis of various cancers, including liver, gastric, and pancreatic cancers [4,5,6,7]. In healthy individuals, CEA levels typically remain below 5 ng/mL, while serum CEA concentrations exceeding 20 ng/mL often indicate the possible presence of cancer [8,9]. Therefore, the rapid and sensitive detection of CEA levels is critical for an accurate early cancer prediction and diagnosis.

Currently, immunoassays are prevalent in clinical tumor marker detection due to their high specificity and sensitivity [10,11]. The existing methods include colorimetric immunoassays [12], fluorescence immunoassays [13], enzyme-linked immunosorbent assays [14], and electrochemiluminescence immunoassays [15,16,17]. While these methods are effective, they require significant reagent volumes, specialized equipment, and skilled personnel, which challenge the need for a rapid clinical diagnosis. Therefore, there is an urgent need to develop convenient detection methods for CEA detection to better serve clinical needs. Label-free electrochemical immunosensors have emerged as a promising solution to overcome the above issues in tumor biomarker monitoring due to their high precision, simplicity, miniaturization, and excellent biocompatibility [18,19]. Label-free electrochemical immunosensors directly monitor changes in current intensity during antigen–antibody interactions, eliminating the need for labeling procedures and avoiding interference from labeling agents [20,21]. The rational design of advanced nanomaterials for electrode modification is crucial in constructing high-performance, label-free electrochemical immunosensors.

With the development of nanomaterial synthesis technology, the application of nanomaterials in the field of electroanalytical sensing, such as metal nanoparticles [22], metal oxide nanoparticles [23], carbon materials [24,25,26], porous silica materials [27,28,29,30], and so on, is garnering increasing attention. As a widely used metal nanoparticle, gold nanoparticles (AuNPs) have been employed as an electrode-modified material to promote electrode analytical performance by enhancing electrode conductivity and improving electron transfer to the sensing interface [31,32,33,34]. In addition, AuNPs are regarded as a kind of ideal material for anchoring biological recognition elements due to their low toxicity and outstanding biocompatibility [35]. Therefore, developing a simple method for the preparation of AuNPs with a nanoscale size on the electrode surface is of great importance.

**Scheme 1:**
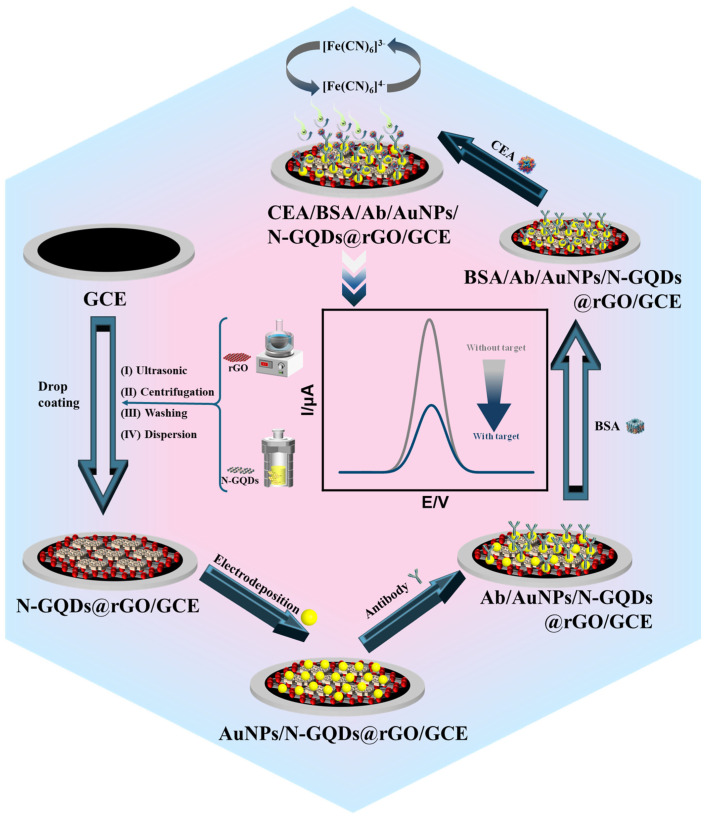
Illustration of the construction of a AuNPs/N-GQDs@rGO-based label-free electrochemical immunosensor for the sensitive detection of CEA.

As a kind of two-dimensional (2D) planar carbon material, reduced graphene oxide nanosheets (rGOs) are extensively utilized as the support material for the growth of metal nanoparticles (e.g., AuNPs, silver nanoparticles, and platinum nanoparticles) [36,37,38] and the porous membrane [39,40,41] to promote the conductivity of electrodes. Researchers have reported on the exploitation of rGO nanosheets for the preparation of AuNPs with the help of protective agents to avoid the aggregation of AuNPs [42]. In this study, hybrid nanocomposites comprising nitrogen-doped graphene quantum dots (N-GQDs) and rGOs were synthesized via the π–π stacking interaction, and they were then employed to grow AuNPs in situ without the need for protective agents using a controllable electrodeposition method (Scheme 1). Such obtained AuNP/N-GQDs@rGO ternary nanocomposites combine the characteristics of three nanomaterials, showing a large surface area, excellent solubility, good conductivity and catalytic activity, a simple fabrication process, and notable stability. Firstly, belonging to zero-dimensional (0D) carbon materials, N-GQDs have similar sheet-like π–π conjugated structures to rGOs and their size is smaller than 10 nm [43,44], which can act as a spacer to avoid the re-stacking of graphene [45]. Secondly, the doped N heteroatom in NGQDs can improve the conductivity of the overall materials, and, meanwhile, they offer more anchoring sites for the further functionalization of other materials [46]. Thirdly, the electrodeposition of AuNPs on the N-GQDs@rGO surface is convenient without the addition of protective agents, which greatly remains the active sites of AuNPs. The combination of the three materials listed above not only forms the electroactive substrate electrode, but also easily generates the biofunctional sensing interface. When the CEA-specific antibody is covalently modified on the AuNPs/N-GQDs@rGO nanocomposites via Au-NH_2_ or Au-SH interactions, a highly sensitive and selective immunosensing interface is achieved. Furthermore, the AuNPs/N-GQDs@rGO-based immunosensor can be applied to accurately determine CEA in fetal bovine serum samples, providing an alternative strategy for the clinical screening of cancer.

## 2. Results and Discussion

### 2.1. The Morphology and Structure of AuNPs/N-GQDs@rGO Composites

**Figure 1:**
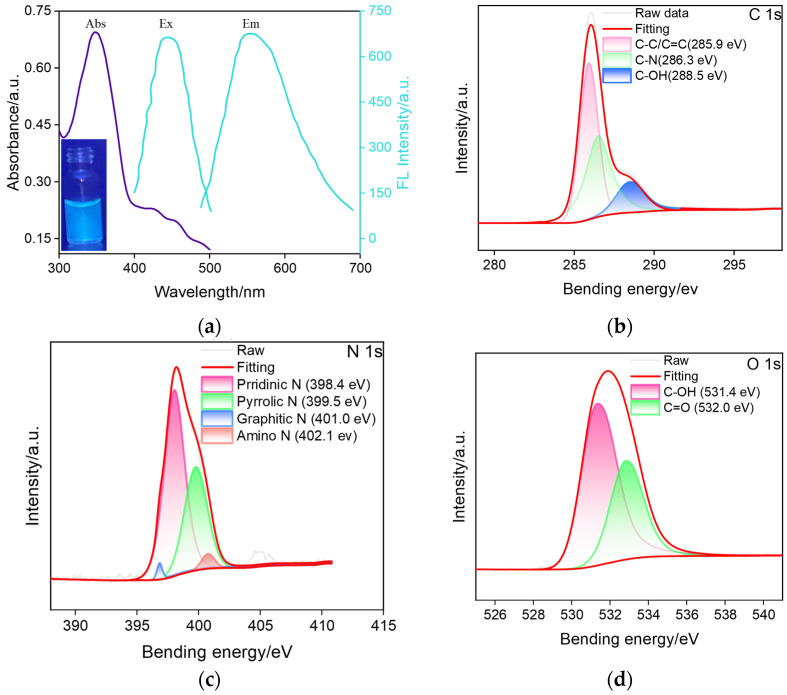
(**a**) UV−Vis (Abs) and fluorescence spectra (*E*_x_, *E*_m_) of N-GQDs. High-resolution C 1s (**b**), N 1s (**c**), and O 1s spectra (**d**) of N-GQDs.

N-GQDs were successfully synthesized using 1-aminopyrene as a precursor and ammonia as a dopant through a one-step hydrothermal method. Due to the quantum confinement effect, the N-GQDs exhibit fluorescence (inset in Figure 1a), which distinguishes them from 2D graphene. The maximum emission wavelength of the N-GQDs is 520 nm when the excitation wavelength is set to 465 nm (Figure 1a). In addition, a distinct ultraviolet absorption peak of the N-GQDs is observed at approximately 350 nm, which is attributed to the n→π* transition of the C=O. The elemental and chemical compositions of the N-GQDs were further analyzed by X-ray photoelectron spectroscopy (XPS) with results shown in Figure 1b–d. The XPS analysis reveals that the N-GQDs are primarily composed of carbon (C), oxygen (O), and nitrogen (N) elements. In the high-resolution C 1s spectrum, peaks at binding energies of 285.9 eV, 286.3 eV, and 288.5 eV are assigned to the C=C, C-N, and C–OH bonds, respectively (Figure 1b) [47,48]. The N 1s spectrum confirms the presence of amino, graphitic, and pyrrolic nitrogen (Figure 1c) [49]. The O 1s spectrum shows characteristic peaks at binding energies of 531.4 eV and 532.0 eV, corresponding to the C–O and C=O bonds, respectively (Figure 1d). All above data verify the successful synthesis of N-GQDs.

**Figure 2:**
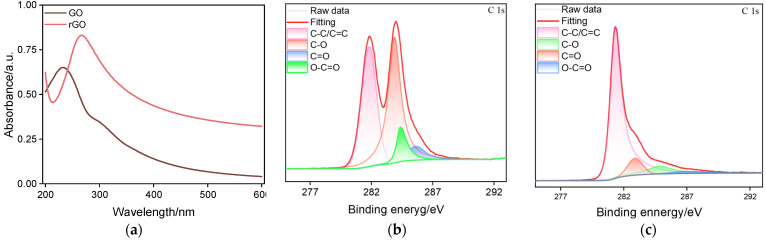
(**a**) UV−Vis spectra of GO (Brown) and rGO (Red). High-resolution C1s XPS spectra obtained on (**b**) GO and (**c**) rGO.

rGO was synthesized via the reduction of GO and was characterized using UV–Vis spectroscopy and XPS. As shown in Figure 2a, the UV–Vis spectrum of GO exhibits a pronounced peak at around 230 nm, which is attributed to the π→π* transitions of conjugated C=C bonds and further indicates the presence of aromatic structures. Additionally, a shoulder peak observed near 300 nm arises from oxygen-containing groups [50]. In comparison with GO, rGO shows a characteristic red shift in the adsorption peak to 260 nm, while the shoulder peak at 300 nm diminishes, indicating that rGO is successfully reduced from GO and the π–π conjugated structure of graphene nanosheets is restored. Figure 2b,c displays the high-resolution C 1s spectra of GO and rGO. Four typical peaks are identified at binding energies of 285.4 eV, 287.2 eV, 288.5 eV, and 289.0 eV in the C 1s spectrum of GO, which is assigned to the C–C/C=C in aromatic rings, C–O, C=O, and O-C=O, respectively [51,52]. By contrast, the C 1s spectrum of rGO reveals an increase in the C–C/C=C intensity and a marked reduction in oxygen-containing functional groups signals, suggesting the effective reduction in GO and the successful formation of rGO [53].

**Figure 3:**
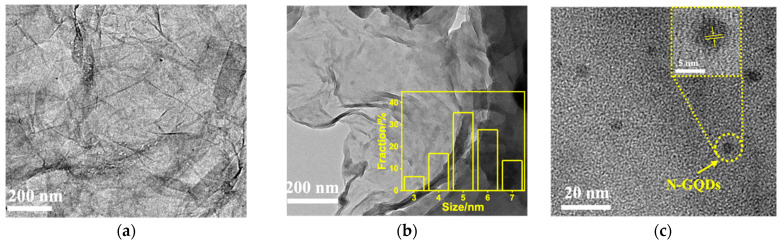
TEM images of rGO (**a**) and N-GQDs@rGO (**b**). Inset in (b) is size distribution of NGQDs N-GQDs@rGO complex. (**c**) HRTEM image of N-GQDs@rGO. Insets is the high-magnification HRTEM image of N-GQDs in N-GQDs@rGO complex.

**Figure 4:**
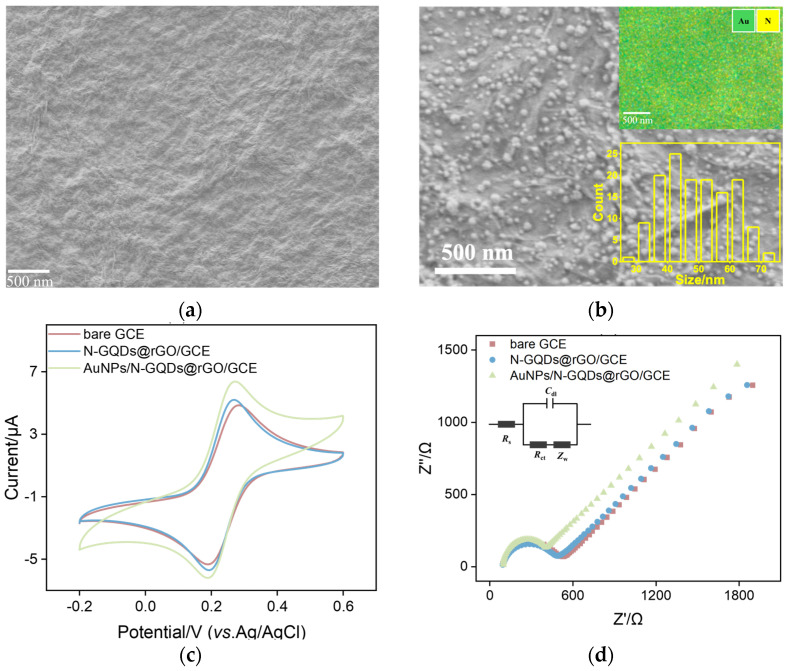
SEM images of N-GQDs@rGO (**a**) and AuNPs/N-GQDs@rGO (**b**). The inset in (**b**) is the EDX mapping of Au and N elements (**top**) and size distribution of AuNPs (**bottom**). (**c**) CV curves obtained at the bare GCE, N-GQDs@rGO/GCE, and AuNPs/N-GQDs@rGO/GCE in a KHP (0.05 M) solution containing Fe(CN)_6_^3−^ (0.5 mM). (**d**) EIS plots obtained at the bare GCE, N-GQDs@rGO/GCE, and AuNPs/N-GQDs@rGO/GCE in KCl (0.1 M) containing Fe(CN)_6_^3/4−^ (2.5 mM). The frequency range for EIS measurements was from 0.1 Hz to 100 kHz, with a perturbation amplitude of 5 mV.

The morphology of the N-GQD@rGO nanocomposites was analyzed using transmission electron microscopy (TEM). Figure 3a illustrates that a wrinkled interconnected network formed at the edges of the rGO nanosheets. The TEM image of N-GQD@rGO reveals that the N-GQDs with an average diameter of 5.3 ± 0.3 nm (47 samples) are uniformly distributed onto the rGO structures (Figure 3b). The lattice spacing of the N-GQDs is 0.28 nm, which corresponds to that of graphene (100) planes (Figure 3c). These N-GQDs can act as the spacer to prevent the restacking of rGO nanosheets, leading to the stable N-GQDs@rGO nanocomposites. The N-GQDs@rGO dispersion shows much better dispersibility compared with the rGO, which arises from the spacer role of the N-GQDs (Figure S1). Figure 4a,b compares the scanning electron microscope (SEM) image of N-GQDs@rGO and AuNPs/N-GQDs@rGO. As seen, the N-GQDs@rGO composites exhibit a wrinkled texture. After the electrodeposition of AuNPs, lots of AuNPs with an average diameter of approximately 50 ± 11 nm (140 samples) are anchored on the N-GQDs@rGO substrate, enhancing the dispersion and stability under the tested conditions. Generally, the smaller AuNPs (<20 nm) may offer higher surface area, but they tend to aggregate during electrochemical procedures. In addition, the Au element of AuNPs and the N element of N-GQDs are found at the energy-dispersive X-ray (EDX) mapping data (inset of Figure 4b), demonstrating the successful preparation of the AuNPs/N-GQDs@rGO ternary nanocomposite.

To further confirm the stepwise fabrication procedure of the AuNPs/N-GQDs@rGO, cyclic voltammetry (CV) and electrochemical impedance spectroscopy (EIS) techniques were employed. Figure 4c,d presents the CV (c) and Nyquist (d) curves of the bare GCE, N-GQDs@rGO/GCE, and AuNPs/N-GQDs@rGO/GCE in a solution containing ferricyanide (Fe(CN)_6_^3−^, 0.5 mM) and ferricyanide/ferrocyanide ([Fe(CN)_6_]^3−^/^4−^, 2.5 mM), respectively. As displayed, the bare GCE exhibits a pair of well-defined redox peaks with a peak-to-peak separation (Δ*E*_p_) of 82 mV corresponding to the electrochemical reaction of Fe(CN)_6_^3−^ and an electron transfer resistance (*R*_ct_) of 376 Ω. Upon modification of N-GQDs@rGO, reduced Δ*E*_p_ (80 mV) and *R*_ct_ (352 Ω) are found at the N-GQDs@rGO/GCE, indicating the increased electroactive surface area and improved conductivity of N-GQDs@rGO nanocomposites. When GCE is modified with AuNPs/N-GQDs@rGO, a significant increase in redox peak currents and a further decreased *R*_ct_ are obtained, which is indicative of the superior electrocatalytic activity of the AuNPs/N-GQDs@rGO ternary composites. These characteristics underscore the significant potential of this ternary composite material for constructing high-performance electrochemical immunosensors for CEA detection.

### 2.2. Electrochemical Characteristics of AuNPs/N-GQDs@rGO-Based Immunosensor

**Figure 5:**
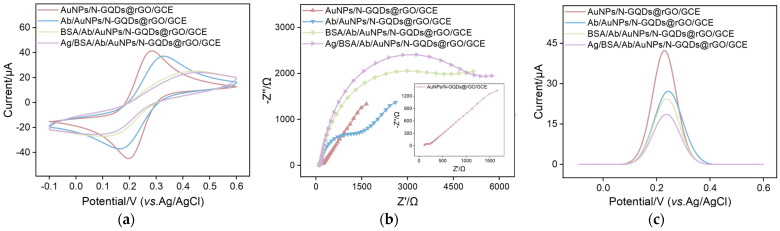
CV (**a**), EIS (**b**) and (**c**) DPV curves on the AuNPs/N-GQDs@rGO/GCE, Ab/AuNPs/N-GQDs@rGO/GCE, and BSA/Ab/AuNPs/N-GQDs@rGO/GCE before and after incubation with CEA (1 ng/mL) in 0.1 M KCl solution containing 2.5 mM Fe(CN)^3−/4−^. The frequency range for EIS measurements was from 0.1 Hz to 100 kHz, with a perturbation amplitude of 5 mV.

In constructing the CEA immunosensor, the AuNPs/N-GQDs@rGO ternary composites were selected as the electrode modification material due to their low toxicity, excellent biocompatibility, and ability to bind antibodies via Au-NH_2_ bonds [54]. CV, EIS, and DPV are effective techniques for observing changes in electrode performance at various stages of modification and the results are shown in Figure 5a–c. As depicted in Figure 5a–c, CV, EIS, and DPV curves of various electrodes in a solution of KCl (0.1 mM) containing [Fe(CN)_6]_^3−^/^4−^ (2.5 mM) demonstrate that, with the successive incubation of CEA-specific antibody (Ab) and BSA, the redox peak currents progressively decrease and *R*_ct_ increases gradually, suggesting the successful fabrication of the AuNPs/N-GQDs@rGO-based immunosensor. Moreover, after incubation with the target CEA (1 ng/mL), the fabricated AuNPs/N-GQDs@rGO-based immunosensor shows a remarkable signal variation, such as the decreased redox currents and the increased *R*_ct_, verifying the feasibility of the developed AuNPs/N-GQDs@rGO-based immunosensor for CEA determination.

### 2.3. Optimization of Experiential Conditions of the AuNPs/N-GQDs@rGO/GCE-Based Immunosensor

**Figure 6:**
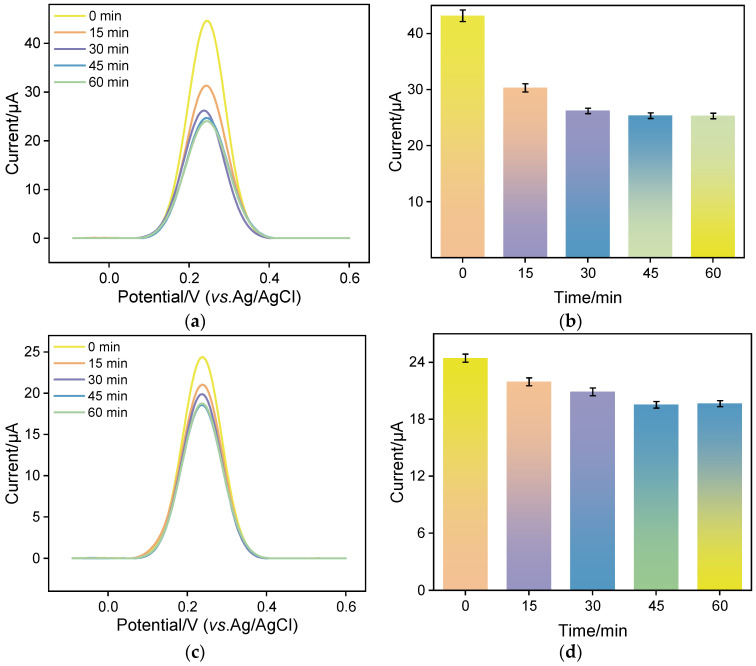
(**a**) DPV curves of AuNPs/N-GQDs@rGO/GCE after incubation with 1 μg/mL of anti-CEA antibody at different incubation times in a 0.1 M KCl solution containing 2.5 mM [Fe(CN)_6_]^3−^/^4−^. (**b**) Dependence of the incubation time for 1 μg/mL anti-CEA antibody on the anodic peak current variation in the Ab/AuNPs/N-GQDs@rGO/GCE. (**c**) DPV curves of AuNPs/N-GQDs@rGO/GCE-based immunosensor after incubation with 100 pg/mL CEA at various incubation times. The tested solution is 0.1 M KCl solution containing 2.5 mM [Fe(CN)_6_]^3−^/^4−^. (**d**) Dependence of the incubation time for 100 pg/mL CEA on the anodic peak current variation in AuNPs/N-GQDs@rGO/GCE-based immunosensor.

To achieve optimal electrochemical responses, parameters influencing the performance of the AuNPs/N-GQDs@rGO/GCE-based immunosensor were systematically investigated, including the antibody’s incubation time and the CEA reaction time. It can be found, in Figure 6a, that, as the reaction time increases, the anodic peak current of [Fe(CN)_6_]^3−^/^4−^ at the Ab/AuNPs/N-GQDs@rGO/GCE gradually decreases. This decrease arises from the following three reasons. First, steric hindrance resulting from the antibody can decrease the mass transport of [Fe(CN)_6_]^3−^/^4−^. Second, due to the increased amount of nonconductive antibodies immobilized on the electrode surface, interfacial resistance enhances and, thereby, decelerates the electron transfer of [Fe(CN)_6_]^3−^/^4−^. Third, charged residues on the antibody may exert the electrostatic effect towards [Fe(CN)_6_]^3−^/^4−^. When the incubation time reaches 30 min, the electrochemical signal remains unchanged, suggesting that antibody immobilization on the AuNPs surface nears saturation (Figure 6b). Therefore, the incubation time for the antibody was selected as 30 min for subsequent studies. Additionally, the effect of the CEA binding time on the detection performance was examined. Figure 6c demonstrates that, when the incubation time of the target CEA reaches 45 min, the electrochemical signal reaches a plateau, indicating that the CEA binding has reached saturation. Consequently, in subsequent experiments, the incubation time for CEA on the AuNPs/N-GQDs@rGO/GCE-based immunosensor was set to 45 min. These optimization steps establish a reliable foundation for further analytical applications.

### 2.4. DPV Performance of the AuNPs/N-GQDs@rGO/GCE-Based Immunosensor

**Figure 7:**
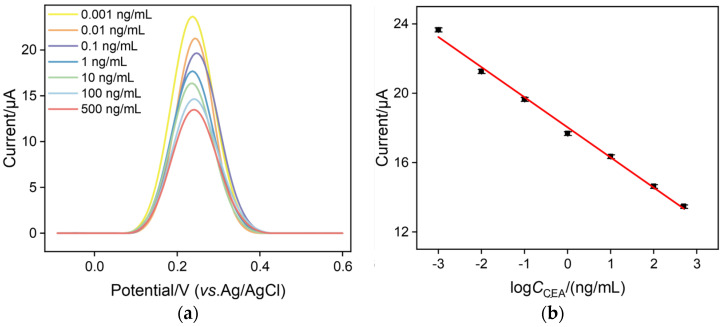
(**a**) DPV curves obtained at the AuNPs/N-GQDs@rGO/GCE-based immunosensor after incubation with different concentrations of CEA in 0.1 M KCl solution containing 2.5 mM [Fe(CN)_6_]^3−^/^4−^. The concentrations of CEA are 1 pg/mL, 10 pg/mL, 0.1 ng/mL, 1 ng/mL, 10 ng/mL, 0.1 μg/mL, and 0.5 μg/mL. (**b**) The calibration curve for the detection of CEA. The error bars represent the standard errors of three measurements.

Under optimal detection conditions, the performance of the constructed AuNPs/N-GQDs@rGO/GCE-based immunosensor for CEA detection was evaluated. Figure 7a displays the DPV signals of the AuNPs/N-GQDs@rGO/GCE-based immunosensor after incubation with varying concentrations of CEA. The results indicate a declining trend in DPV signals with increasing CEA concentration, attributable to the impeded electron transfer effect by the accumulation of immune complexes on the electrode surface. As depicted in Figure 7b, a good linear relationship is observed between the anodic peak current (*I*) and the logarithm of CEA concentration (log*C*_CEA_) over the range of 1 pg/mL to 500 ng/mL, with the regression equation *I* (μA) = −1.73 (± 0.054) log*C*_CEA_ (ng/mL) + 18.0 (± 0.104), and a correlation coefficient (*R*^2^) of 0.995 (RSD < 3%). The limit of detection (LOD), calculated based on a signal-to-noise ratio (S/N = 3), is determined to be 0.13 pg/mL. Table 1 compares the CEA detection performances of our immunosensor with other reported electrochemical immunosensors. As seen, the dynamic linear range obtained at our developed AuNPs/N-GQDs@rGO/GCE-based immunosensor is broader than those of other electrochemical sensors. Although the achieved LOD (0.13 pg/mL) is lower than those at the BSA/Ab/chitosan (CS)-rGO/gold nanodendrites (AuND)/AuNPs/GCE, BSA/Ab/streptavidin/CS/platinum nanoparticles (PtNPs)@rGO@polystyrene nanospheres (PS NSs)/GCE (0.01 pg/mL), BSA/Ab/Au/ZnMn_2_O_4_@rGO/GCE (0.019 pg/mL), Au@CeBi_0.4_O_3.7_ with feather-like structures (CeBi_0.4_O_3.7_-Ab2NFs)/CEA/BSA/Ab1/rGO-Au/GCE (0.12 pg/mL), AuNPs/covalent organic frameworks (COF) TFPB-Thi/Ab2/CEA/BSA/Ab1/COF Tab-Dva/GCE (0.03 pg/mL), and Cu_2_O/PtNPs-Ab2/CEA/BSA/Ab1/AuNPs/GCE (0.03 pg/mL), our AuNPs/N-GQDs@rGO/GCE-based immunosensor simplifies the electrode construction process, offering a convenient method for detecting trace CEA. Above results indicate that our developed AuNPs/N-GQDs@rGO/GCE-based immunosensor exhibits excellent detection performance across a broad concentration range.

### 2.5. Selectivity, Anti-Interference, Reproducibility, and Stability of the AuNPs/N-GQDs@rGO/GCE-Based Immunosensor

**Figure 8:**
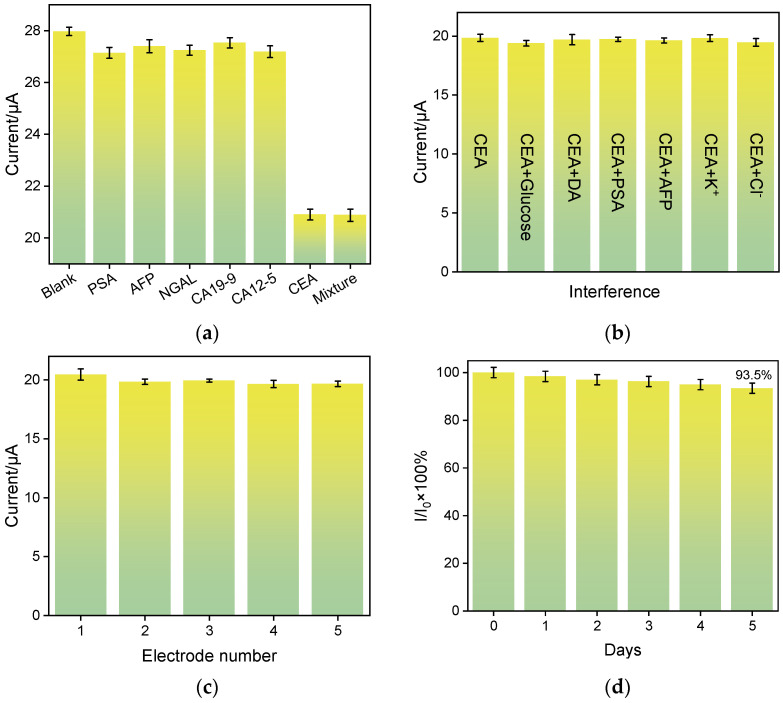
(**a**) Anodic peak currents of AuNPs/N-GQDs@rGO/GCE-based immunosensor after incubation with PSA (10 ng/mL), AFP (10 ng/L), NGAL(10 ng/mL), CA19-9 (100 U/mL), CA125 (1 U/mL), CEA (100 pg/mL), or their mixture in 0.1 M KCl solution containing 2.5 mM [Fe(CN)_6_]^3−^/^4−^. (**b**) Anodic peak currents of AuNPs/N-GQDs@rGO/GCE-based immunosensor after incubation with CEA (100 pg/mL) and other interfering species (1 ng/mL) in a 0.1 M KCl solution containing 2.5 mM Fe(CN)_6_^3−^/^4−^. (**c**) Anodic peak currents of five AuNPs/N-GQDs@rGO/GCE-based immunosensor after incubation with CEA (100 pg/mL) in a 0.1 M KCl solution containing 2.5 mM [Fe(CN)_6_]^3−^/^4−^. (**d**) Relative ratio of andic peak current obtained at the immunosensor after incubation CEA (100 pg/mL) and stored at 4 °C for different days. *I*_0_ and *I* are anodic peak currents obtained at the first and other days.

To verify the selectivity of the AuNPs/N-GQDs@rGO/GCE-based immunosensor, the influence of common tumor biomarkers as potential interferents on CEA detection was investigated, such as prostate-specific antigen (PSA), alpha-fetoprotein (AFP), neutrophil gelatinase-associated lipocalin (NGAL), and carbohydrate antigens 19-9 (CA19-9) and CA12-5. As illustrated in Figure 8a, when these substances were incubated individually with the our immunosensor, the variation in anodic peak current is negligible. Only in the presence of CEA or mixtures containing CEA, the anodic peak current decreases remarkably and their variation is comparable, suggesting the excellent selectivity and anti-interference capacity of our fabricated immunosensor. In addition, the AuNPs/N-GQDs@rGO/GCE-based immunosensor was co-incubated with the additional potential interferents that may coexist with CEA in human serum, including glucose, dopamine, PSA, AFP, K^+^, and Cl^−^. As depicted in Figure 8b, even when the concentration of interferents is ten-fold that of CEA, variations in the DPV current response remains negligible, demonstrating the immunosensor’s superior anti-interference capability. Figure 8c shows the anodic peak currents of five immunosensors after incubation with CEA (100 pg/mL). The DPV signals of these electrodes, measured in [Fe(CN)_6_]^3−^/^4−^ solution, exhibit a low relative standard deviation (RSD) of 1.8%, indicating the excellent reproducibility. The storage stability of the immunosensor incubated with CEA antibodies was also evaluated by incubation with CEA (100 pg/mL) and stored at 4 °C for five days. Figure 8d records the anodic peak currents of the immunosensor within five days and the signal retains 93.5% of its initial value after five days, confirming that the designed immunosensor exhibits commendable storage stability.

### 2.6. Real Sample Analysis

To assess the potential of the developed AuNPs/N-GQDs@rGO/GCE-based immunosensor for practical applications, fetal bovine serum was chosen as a representative real sample, and the concentration of CEA was quantified using the standard addition method. Throughout the experiment, various concentrations of CEA were added to the different simulated samples and the detected results were shown in Table 2. As presented, the recoveries of the immunosensor range from 101% to 103%, with an RSD of less than 3.2%. These data demonstrate the immunosensor’s outstanding reliability and accuracy in detecting real samples, showing a good anti-fouling ability in complex biological matrices.

## 3. Materials and Methods

### 3.1. Chemicals and Materials

CEA, anti-CEA antibodies, alpha-fetoprotein (AFP), prostate-specific antigen (PSA), and fetal bovine serum were obtained from Beijing KeyGen Biotech Co., Ltd. (Beijing, China). A monolayered GO aqueous dispersion (10 mg/g) was sourced from Hangzhou GaoxiTech (Hangzhou, China). Hydrazine hydrate was sourced from Hangzhou Shuanglin Chemical Reagent Co., Ltd. (Hangzhou, China). Various reagents, including potassium ferricyanide (K_3_[Fe(CN)_6_], 99.5%), potassium ferrocyanide (K_4_[Fe(CN)_6_], 99.5%), potassium chloride (KCl, AR), chloroauric acid (HAuCl_4_·3H_2_O, 99.9%), 1-aminopyrene (C_16_H_11_N, 98%), ammonia solution (NH_3_·H_2_O, AR), sodium dihydrogen phosphate (NaH_2_PO_4_·2H_2_O, AR), and disodium hydrogen phosphate (Na_2_HPO_4_·12H_2_O, AR), were obtained from Aladdin Biochemical Technology Co., Ltd. (Shanghai, China). Ethanol (99.8%) was procured from Hangzhou Gaojing Fine Chemical Co., Ltd. (Hangzhou, China). A glassy carbon electrode (GCE, 3 mm in diameter) was acquired from CHI Instrument Co., Ltd. (Shanghai, China). Phosphate buffer solution (PBS) was prepared using Na_2_HPO_4_ and NaH_2_PO_4_. All chemicals employed in this study were of analytical grade and used without further purification. Ultrapure water (18.2 MΩ cm) used in the experiments was obtained from a Milli-Q system (Millipore Company, Burlington, MA, USA).

### 3.2. Characterizations and Instrumentations

The morphologies of rGO and N-GQDs@rGO were examined using a transmission electron microscope (TEM, JEM-2100, JEOL, Musashino, Japan) operating at 200 kV. In addition, the morphologies of N-GQDs@rGO and AuNPs/N-GQDs@rGO were analyzed using a scanning electron microscope (SEM, SU8010, Hitachi, Tokyo, Japan) at 10 kV. Ultraviolet–visible (UV–Vis) absorption spectroscopy was conducted using a UV-2450 spectrometer (Shimadzu, Tokyo, Japan). X-ray photoelectron spectroscopy (XPS) was conducted using a PHI5300 electron spectrometer (PE Ltd., Waltham, MA, USA) equipped with an Mg Kα source (250 W, 14 kV). A conventional three-electrode system was employed: Ag/AgCl served as the reference electrode, platinum wire as the counter electrode, and the modified electrode as the working electrode. An Autolab electrochemical workstation (PGSTAT302N, Metrohm, Switzerland) was utilized to conduct all electrochemical experiments, namely, cyclic voltammetry (CV), differential pulse voltammetry (DPV), and electrochemical impedance spectroscopy (EIS). The scan rate for CV tests was set to 50 mV/s, and the parameters for DPV tests were defined as follows: step potential of 0.005 V, modulation amplitude of 0.025 V, modulation time of 0.05 s, and interval time of 0.2 s. The frequency range for EIS measurements was from 0.1 Hz to 100 kHz, employing a perturbation amplitude of 5 mV.

### 3.3. Preparation of rGO

rGO was synthesized via a modified chemical reduction method [64,65]. Briefly, 8 mL of GO (1 mg/mL) solution was added to 32 mL of water. The mixture was ultrasonicated for approximately 24 h to ensure the thorough dispersion of GO in the water. Once the GO solution was uniformly dispersed, 135 μL of ammonia solution (25 wt%) and 14 μL of hydrazine hydrate (40 wt%) were sequentially added. The mixture was then stirred in a water bath at 85 °C for 3 h. Upon completion of the reaction, the solution was centrifuged at 3000 rpm for 30 min to yield a homogeneous rGO dispersion.

### 3.4. Preparation of N-GQDs

N-GQDs were synthesized by one-step hydrothermal synthesis method [66,67,68]. Initially, 40 mg of 1-aminopyrene was dissolved in 20 mL of ammonia solution (0.4 M). The resulting mixture was transferred into a polytetrafluoroethylenelined autoclave (50 mL capacity) and subjected to a high-pressure reaction at 200 °C for 8 h. Upon completion of the reaction, the solution was filtered through a 0.22 μm membrane to remove large particles. Subsequently, the filtrate was dialyzed using a dialysis bag with a molecular weight cut-off of 500 Da for 24 h to remove the unreacted small molecules. Finally, the dialysate was freeze-dried to obtain the NGQDs.

### 3.5. Preparation of AuNPs/N-GQDs@rGO/GCE

rGO (1 mL) and N-GQDs (200 μL) dispersions were mixed, ultrasonicated for 2 h, centrifuged (12,000 rpm, 30 min), and washed with ethanol/water to obtain N-GQDs@rGO. The glassy carbon electrode (GCE) was polished with 0.3 and 0.05 µm alumina slurry, nitric acid (50%), anhydrous ethanol, and ultrapure water sequentially to remove residual alumina particles. After sonication, the GCE was dried with nitrogen gas for subsequent use. Subsequently, 4 µL of the N-GQDs@rGO composite solution was drop-casted onto the GCE surface, and dried at 60 °C to obtain the N-GQDs@rGO-modified GCE (denoted as N-GQDs@rGO/GCE). AuNPs were electrodeposited by immersing the modified GCE in a 0.5 mM HAuCl_4_ solution at −0.5 V for 2 s, followed by rinsing and storage at 4 °C.

### 3.6. Construction of Label-Free Electrochemical Immunosensors BSA/Ab/AuNPs/N-GQDs@rGO/GCE

4 μL anti-CEA antibody (Ab, 1 μg/mL) was drop-casted onto the surface of the AuNPs/N-GQDs@rGO/GCE and incubated at 4 °C for 45 min to ensure stable immobilization of Ab on the GCE surface. Following incubation, the resulting electrode was gently rinsed with PBS (0.1 M, pH 7.0) to remove unbound substances. Subsequently, to block the active sites on the electrode surface and reduce nonspecific adsorption, 4 μL BSA (0.1 mg∙mL^−1^) was coated on the surface of Ab/AuNPs/N-GQDs@rGO/GCE at 4 °C for 60 min. After rinsing with PBS (0.1 M, pH 7.0), the immunosensor was achieved, designated as the BSA/Ab/AuNPs/N-GQDs@rGO/GCE, and stored at 4 °C.

## 4. Conclusions

In summary, an AuNPs/N-GQDs@rGO ternary nanocomposite was successfully pre-pared and employed as a kind of electrode-modified material on the GCE surface to construct a sensitive electrochemical immunosensor for enhanced electrochemical detection performance for CEA. Such AuNPs/N-GQDs@rGO nanocomposites display a large surface area, good conductivity and high electrocatalytic capacity, abundant functional sites, facile modification capacity, and robust stability. By a simple covalent modification of the CEA-specific antibody on the conductive AuNPs/N-GQDs@rGO/GCE surface, the proposed AuNPs/N-GQDs@rGO/GCE-based electrochemical immunosensor enables the selective and sensitive determination of CEA with a broad detection linear range and a satisfactory LOD of 0.13 pg/mL. Moreover, the direct and accurate analysis of CEA in fetal bovine serum samples has been realized by the developed electrochemical immunosensor, which can be expected to aid in the preparation of various tumor biomarkers and further integrated with smart chips for significant applications in the clinical diagnosis of diseases.

## Figures and Tables

**Table 1:** Comparison with the other reported electrochemical immunosensors to CEA detection.

Sensing Interface	Linear Range (ng∙mL^−1^)	LOD (pg∙mL^−1^)	Analysis Method	Construction Step	Refs.
BSA/Ab/CS ^1^-rGO/AuND ^2^/AuNPs/GCE	0.0001–10	0.02	DPV	6	[55]
BSA/Ab/streptavidin/CS/PtNPs ^3^@rGO@PS NSs ^4^/GCE	0.05–70	0.01	DPV	6	[56]
BSA/Ab/Au/ZnMn_2_O_4_@rGO/GCE	0.01–50	0.019	DPV	5	[57]
BSA/Au-pMCP ^5^-Ab/Au/3D-G ^6^/GCE	0.5–200	0.31	SWV ^13^	5	[58]
BSA/Ab/AuNPs@Ir-Zr-MOL ^7^/GCE	0.001–100	0.2	ECL ^14^	4	[59]
Au@CeBi_0.4_O_3.7_-Ab2NFs ^8^/CEA/BSA/Ab1/rGO-Au/GCE	0.01–100	0.12	DPV	7	[60]
AuNPs/COF ^9^TFPB-Thi/Ab2/CEA/BSA/Ab1/COF Tab-Dva/GCE	0.11–80	0.03	DPV	7	[61]
Cu_2_O/PtNPs-Ab2/CEA/BSA/Ab1/AuNPs/GCE	0.0001–80	0.03	CA ^15^	6	[62]
Apt ^10^/GNPs ^11^/MOF ^12^(801)/rGO/GCE	2.5–250	800	EIS	4	[63]
BSA/Ab/AuNPs/N-GQDs@rGO/GCE	0.001–500	0.13	DPV	5	This work

^1^: CS: chitosan; ^2^: gold nanodendrites; ^3^: platinum nanoparticles; ^4^: polystyrene nanospheres; ^5^: poly-m-cresol purple; ^6^: three-dimensional graphene; ^7^: metal organic layers; ^8^: CeBi_0.4_O_3.7_ with feather-like structures; ^9^: covalent organic frameworks. ^10^: aptamer; ^11^: gold nanoparticle; ^12^: metal–organic framework; ^13^: square wave voltammetry; ^14^: enhanced chemiluminescence; ^15^: CA: chronoamperometry.

**Table 2:** Detection of CEA in fetal bovine serum samples.

Sample	Added ^b^ (ng/mL)	Found (ng/mL)	RSD (%, *n* = 3)	Recovery (%)
	0.0100	0.0103	3.2	103
Serum ^a^	0.100	0.101	2.8	101
	10.0	10.1	2.5	101

^a^: Samples with the added CEA were diluted by a factor of 50 using 0.1 M PBS (pH 7.4); ^b^: The concentration of CEA presented in Table 2 reflects the values after dilution.

## Data Availability

The data presented in this study are available upon request from the corresponding author.

## Supplementary Materials

The following supporting information can be downloaded at: https://www.mdpi.com/article/10.3390/molecules30061347/s1, Figure S1: Photograph of rGO and N-GQDs@rGO dispersions after the storage of three months.
