# Supplementary material for: In Situ Growth of Au NPs on Nitrogen-Doped Graphene Quantum Dots Decorated Graphene Composites for the Construction of an Electrochemical Immunosensor and Its Application in CEA Detection
Source: Molecules. 2025 Mar 17;30(6):1347. doi: 10.3390/molecules30061347 (PMC11944492; doi:10.3390/molecules30061347)
Supplement: Supplementary file 1 [file molecules-30-01347-s001.zip › molecules-3384879-supplementary.pdf]

Supporting Information to

# In Situ Growth of Au NPs on Nitrogen-Doped Graphene Quantum Dots Decorated Graphene Composites for the Construction of an Electrochemical Immunosensor and Its Application in CEA Detection

Zhengzheng Yan, Lujie Wang and Fei Yan

## Table of Content

S1. Photograph of rGO and N-GQDs@rGO dispersions

## S1. Photograph of rGO and N-GQDs@rGO dispersions

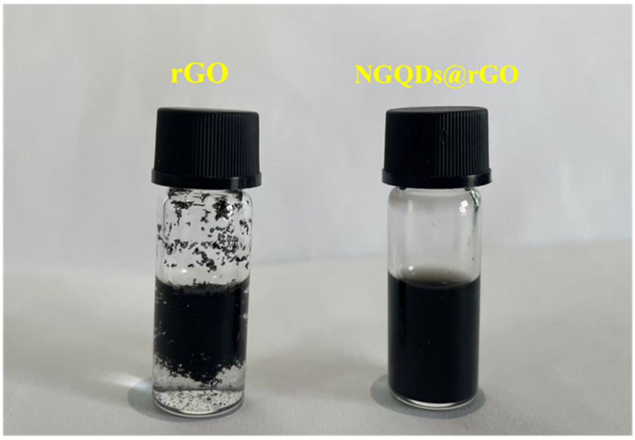


**Figure S1.** Photograph of rGO and N-GQDs@rGO dispersions after the storage of three months.
